# Radiocarbon Dating of an Olive Tree Cross-Section: New Insights on Growth Patterns and Implications for Age Estimation of Olive Trees

**DOI:** 10.3389/fpls.2017.01918

**Published:** 2017-11-10

**Authors:** Yael Ehrlich, Lior Regev, Zohar Kerem, Elisabetta Boaretto

**Affiliations:** ^1^D-REAMS Radiocarbon Laboratory, Kimmel Center for Archaeological Science, Scientific Archaeology Unit, Weizmann Institute of Science, Rehovot, Israel; ^2^The Institute of Biochemistry, Food Science and Nutrition, The Robert H. Smith Faculty of Agriculture, Food and Environment, The Hebrew University of Jerusalem, Rehovot, Israel

**Keywords:** olive, annual rings, radiocarbon, tree age estimation, tree growth

## Abstract

The age of living massive olive trees is often assumed to be between hundreds and even thousands of years. These estimations are usually based on the girth of the trunk and an extrapolation based on a theoretical annual growth rate. It is difficult to objectively verify these claims, as a monumental tree may not be cut down for analysis of its cross-section. In addition, the inner and oldest part of the trunk in olive trees usually rots, precluding the possibility of carting out radiocarbon analysis of material from the first years of life of the tree. In this work we present a cross-section of an olive tree, previously estimated to be hundreds of years old, which was cut down *post-mortem* in 2013. The cross-section was radiocarbon dated at numerous points following the natural growth pattern, which was made possible to observe by viewing the entire cross-section. Annual growth rate values were calculated and compared between different radii. The cross-section also revealed a nearly independent segment of growth, which would clearly offset any estimations based solely on girth calculations. Multiple piths were identified, indicating the beginning of branching within the trunk. Different radii were found to have comparable growth rates, resulting in similar estimates dating the piths to the 19th century. The estimated age of the piths represent a *terminus ante quem* for the age of the tree, as these are piths of separate branches. However, the tree is likely not many years older than the dated piths, and certainly not centuries older. The oldest radiocarbon-datable material in this cross-section was less than 200 years old, which is in agreement with most other radiocarbon dates of internal wood from living olive trees, rarely older than 300 years.

## Introduction

Olive trees (*Olea europaea*) are able to survive for many years, potentially representing a valuable source of information for dendrochronology. Moreover, olive wood remains from ca. 4500 BC are frequently found in archeological contexts in the eastern Mediterranean (Galili et al., 1989; Liphschitz et al., 1991; Epstein, 1993; Gibson and Rowan, 2006). However, these frequent finds are rarely utilized, as the identification of annual rings is problematic (Crivellaro and Schweingruber, 2013) due to asymmetric cambial growth (Terral and Arnold-Simard, 1996; Luz et al., 2014) and has even been deemed impossible due to intra-annual density fluctuations (IADFs) which could not be differentiated from true rings (Cherubini et al., 2013). IADFs are formed as the wood density is abruptly altered due to fluctuations in water availability, as growth during the growing season is halted and subsequently restarted (De Micco et al., 2012, 2016).

The process of wood formation, or xylogenesis, occurs at the vascular cambium which is subject to molecular control able to respond to environmental conditions (Plomion et al., 2001; Fromm, 2013; Ye and Zhong, 2015). In temperate climates, trees are able to endure the cold season by dormancy of cambial activity, resulting in earlywood and latewood (Cherubini et al., 2003; Fromm, 2013). Earlywood is formed early in the growing season, and is characterized by larger vessels, while latewood is formed near the end of the growing season, when unfavorable environmental conditions begin, and vessels are typically smaller and more dense. However, in the Mediterranean, which is characterized by hot and dry summers and cold and wet winters, trees must be able to endure both drought stress and cold stress. The vascular cambium in olive trees was reported to be active during two main periods (Liphschitz and Lev-Yadun, 1986; Drossopoulos and Niavis, 1988). Water availability was shown to have a positive effect on girth (Terral and Durand, 2006; López-Bernal et al., 2010) and vessel size in olive wood, with earlywood and latewood detected in rainfed trees but not in irrigated ones (Rossi and Sebastiani, 2014).

The trunk of the olive tree consists of independent vascular systems connecting different scaffolds to their individual root system. The cambial activity rate at the meeting regions between these segments as well as the differences in development between scaffolds causes the characteristic indentations in diameter observed in older olive tree trunks (Lavee, 1996).

The inner and oldest part of the wood usually decays in older olive trees, making it impossible to identify the true center of origin. These processes also leave no material to allow the exact dating of the ancient trees (Lavee, 1996). Direct dating of living olive trees can only be done on the existing wood which is closest to the hypothetical center, which has rarely been radiocarbon dated to older than 200–300 years (Lavee, 1996). Recently, however, wood from live olive trees in Jerusalem has been radiocarbon dated to 600–700 years old (Bernabei, 2015). Extrapolating the age of the original pith has been based on the rate of increase in girth per year, combined with the current girth of the trunk (Arnan et al., 2012; Bernabei, 2015).

In this work, we explore the chronological pattern of xylem deposition in olive wood from a modern olive tree which grew in northern Israel, utilizing radiocarbon. Higher resolution dating calibration has been applicable for samples from the last ∼60 years, due to nuclear tests carried out which dramatically increased ^14^C levels in the atmosphere over a short period of time (the “bomb peak”) (Hua et al., 2013). Here we report the radiocarbon concentration measured at various points sampled from the transverse section of olive wood and explore its significance for understanding olive wood growth patterns and for estimation of the age of the tree.

## Materials and Methods

### Study Area and Sampling Procedure

**FIGURE 1:**
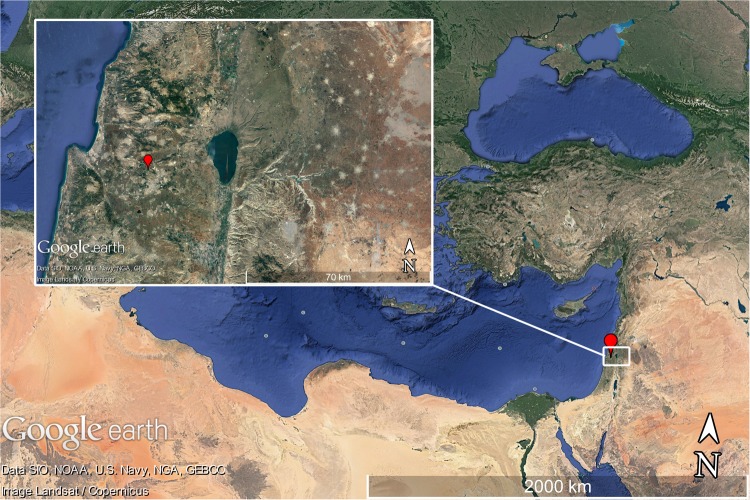
Larger map of the Mediterranean (scale bar of 2000 km); inset: map of the sampling region in northern Israel, marking the location of the site, Zippori, in red (scale bar of 70 km). Maps generated by Google Earth.

A trunk transverse section from an olive (*O. europeae*) tree was obtained for research by one of the authors (ZK), from the site of Zippori (32°45′24.1′′N 35°16′50.9′′E, 195m) in northern Israel during 2013 (**Figure 1**). The tree had died several years prior to cutting it down, and presented an apparent complete trunk. The cross-section was polished gradually to 1000 grit grade: a belt sander (Makita #9404) was used with five grades of grit (24, 40, 80, 150, 320) followed by polishing with a random orbit sander (Makita #BO5041) with four grades of grit (400, 600, 800, and 1000). Samples for α-cellulose extraction were obtained using a Dremel drill (8000 model, 2.8 mm drill bit), and the resulting saw dust was collected from each point.

### Dendrochronological Methods

Tree rings were visually identified using a stereo microscope (M80, Leica) and their widths were measured to the nearest 0.001 mm using a sliding micrometer stage (“TA” measurement system, Velmex, Inc.) and the Tellervo dendrochronological analysis package (Brewer, 2014).

### α-Cellulose Extraction

Wood sawdust was placed in pre-baked 16 mm × 125 mm borosilicate glass test tubes. All glassware, including tubes and Pasteur pipettes, was pre-baked (1 h at 450°C) to eliminate organic contamination. The tops of the tubes were stuffed with meshed glass wool. Acid-base-acid (ABA) pretreatment was carried out: samples were treated with aliquots of 5 ml 1N HCl for 1 h; washed with DDW; treated with 5 ml of 0.1N NaOH for 1 h; washed with DDW and finally treated with 5 ml 1N HCl for 1 h in a water bath at 70°C to remove any carbon that may have adhered during the previous alkaline treatment. Immediately following the ABA pretreatment, holocellulose was extracted using a modification to a variation suggested by Southon and Magana (2010) of the Jayme-Wise method: 2.5 ml of 1N HCl and 2.5 ml of 1M NaClO_2_ were added to the samples which were then transferred to a water bath at 70°C and left overnight. For samples which required further bleaching, the treatment in 2.5 ml of 1N HCl and 2.5 ml of 1M NaClO_2_ was repeated until all samples became white. After bleaching, the samples were washed with DDW. For the extraction of α-cellulose, the samples were treated with 6 ml of 5N NaOH for 1 h, followed by washing with DDW and subsequently treated for 1 h with 5 ml of 1N HCl in a water bath at 70°C. The samples were then washed with DDW until reaching a neutral pH, and dried in an oven at 100°C.

### Radiocarbon Dating

Between 2 and 4 mg of α-cellulose were weighed into pre-baked (1 h at 900°C) quartz tubes containing 200 mg CuO and oxidized to CO_2_ in a vacuum line at 900°C for 3 h. CO_2_ pressure known to eventually result in ∼1 mg carbon was transferred from each sample into tubes containing 1 mg of activated Co for graphitization. ^14^C content determination on the resulting graphite was carried out at the Dangoor Research Accelerator Mass Spectrometry (D-REAMS) laboratory at the Weizmann Institute. All calculated ^14^C ages were corrected for isotopic fractionation based on the stable carbon isotope ratio (δ^13^C value, as measured by the AMS). Calibrated ages in calendar years have been obtained from the NHZ2 calibration curve (Hua et al., 2013) using OxCal v 4.2 (Bronk Ramsey, 2009) or CALIBomb (Reimer et al., 2004).

## Results

### Growth Ring Count Compared with Radiocarbon Dates

In total, 11 samples were taken for radiocarbon dating from a cross-section of a whole section from an olive tree which was cut down a few years after the tree had died, from the site of Zippori in northern Israel. The section was first analyzed visually under a stereo microscope, and putative annual rings were counted.

**FIGURE 2:**
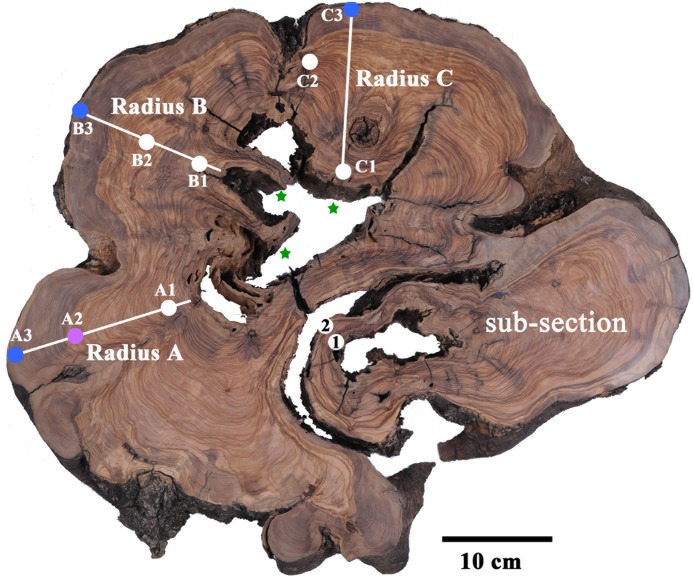
Transverse section of olive tree trunk, cut down in 2013, several years after the tree had died. Sampling points marked on cross-section, color coded to pre-1955 (white); 1970–1979 (magenta); 2000–2009 (blue). Stars mark the presumed location of original piths. Note the bottom right nearly separate sub-section, which was found to be younger than the points A1, B1 and C1, and was therefore not considered in tree age calculation.

In agreement with previous reports, the radiocarbon dates were generally not matched with the number of rings counted (Cherubini et al., 2013). Radius A was sampled at putative rings 1, 26–29, and 77, as counted from the bark (points A3, A2, and A1, respectively, in **Figure 2**). As the tree had been dead for a few years before cutting it in 2013, the outer rings were expected to be within a few years from this date. It should be noted that the latest date in the current calibration available (Hua et al., 2013) is the end of 2009. Thus, we will consider samples dated to 2009 as between 2009 and 2013.

Point A3 was dated to 2002–2004. Sample A2 from rings 26–29, would be expected to give a date about 30 years prior, assuming correct ring count and the continuous formation of annual rings, and indeed 1974–1976 was found to be a possible date for this sample (**Table 1** and Supplementary Table S1). The next sample (point A1 in **Figure 2**) was from ring 77, near the decomposed pith region, which was expected to give a date roughly 80 years before the early 2000s, i.e., ∼1920. The closest possible radiocarbon date for this sample is 1936–1952, which is the date range assigned for this sample. The next dates possible for this sample are 1668–1805, which would require missing a few hundred rings, while identifying only 77 rings. This is an unlikely scenario, as this error range is many times higher than previously reported for counting rings in olive wood (Cherubini et al., 2013).

**Table 1:** Ring count and corresponding radiocarbon date range (±1σ).

Radius	Sample	Ring no.	Range expected	^14^C Cal. date	Likely range
	number	(from bark)	from ring count	measured (± 1σ)	(year CE)
A	A1	77	1920–1930	1668 (12.7%) 1682	**1936–1952**
				1736 (19.3%) 1759	
				1760 (19.0%) 1781	
				1797 (6.1%) 1805	
				**1936 (9.9%) 1947**	
				**1951 (1.2%) 1952**	
	A2	26–29	1970–1980	**1962 (11.3%) 1962**	**1962–1976**
				**1962 (11.3%) 1962**	
				**1974 (24.9%) 1975**	
				**1975 (32.0%) 1976**	
	A3	1	2000–2009	**2002 (68.2%) 2004**	**2002–2004**
B	B1	60	1940–1950	1698 (19.8%) 1722	**1879–1916**
				1816 (13.9%) 1834	
				**1879 (34.3%) 1916**	
				1954 (0.2%) 1955	
	B2	36	1960–1970	1699 (2.6%) 1703	**1954–1955**
				1706 (12.1%) 1720	
				1818 (11.4%) 1833	
				1880 (41.3%) 1915	
				**1954 (0.7%) 1955**	
	B3	1	2000–2009	1957 (2.6%) 1957	**2003–2005**
				**2003 (5.6%) 2003**	
				**2003 (59.9%) 2005**	
C	C1	–	–	1700 (0.7%) 1701	**1881–1915**
				1707 (12.0%) 1719	
				1819 (2.5%) 1823	
				1825 (6.6%) 1833	
				**1881 (45.7%) 1915**	
				1954 (0.7%) 1955	
	C2	–	–	1892 (64.7%) 1907	**1954-1955**
				**1954 (3.5%) 1955**	
	C3	1	–	**2002 (65.2%) 2004**	**2002–2004**
				**2004 (3.0%) 2004**	

*For radius C, the ages are estimated based on the chronological order of the samples. Bold values indicate the most likely dates for samples from among the possible calibrated radiocarbon dates presented in column 5, taking into consideration the putative ring count*.

In radius B the outermost wood was dated to 2003–2005 (point B3 in **Figure 2**), while at a distance of 36 putative rings inward toward the pith, point B2 (**Figure 2**) was dated to 1954–1955, indicating a deficit of about 20 rings. Point B1, closest to the decayed pith was 24 putative rings away from point B2, thus expected to date to ∼1931 (1955–24 = 1931). However, point B1 was dated to 1879–1916, as the latest possible date after 1955 (Supplementary Table S1). In this case too there was a deficit in the number of rings which were visually identified, compared with the dates measured with radiocarbon.

Radius C includes irregular rings due to a notch, and therefore the rings across this radius were not measured as they do not comprise a linear sequence between the pith (nearest point: C1) and the bark (nearest point: C3). However, a sample was taken offset to the radius, between the pith and bark (point C2 in **Figure 2**) to obtain a sequence of three time points. Point C3 was dated to 2002–2004, and point C1 had a wide range of possible dates. As 1954–1955 could be ruled out for point C1, since the latest possible date for the chronologically later point C2 was also 1954–1955, the next possible date range for point C1 was 1881–1915, which is similar to point B1. Earlier dates of 1819–1833 could also be possible, however, the date range of 1700–1719 seems unreasonable based on comparison to other points on the cross-section, as well as on the relative distance and growth pattern between the points.

Points 1 and 2 from a nearly independent segment in the cross-section (**Figure 2**) were both estimated to give very similar dates, as the growth pattern indicates a distance of only five or six rings difference, assuming no IADFs were counted in between. The calibrated radiocarbon dates (Supplementary Table S1) indicate both these samples may date to between 1954 and 1955, a more likely scenario given the growth pattern, than is the option of nearly 50 or more than 200 years difference between the samples. As this segment is somewhat structurally independent of the rest of the trunk and appears to be younger than the main section, it will not be further utilized in the evaluation of the age of the tree.

### Tree Age Determination by Extrapolation

Estimation of the age of the olive tree pith was carried out by extrapolation based on growth rates (as in Arnan et al., 2012; Bernabei, 2015). Rates were calculated between numerous points along three radii in the trunk cross-section from Zippori (**Table 2**). The distance between two dated points was measured in centimeters, and growth rate (cm per year) was thus calculated. This growth rate value was then used to calculate the theoretical number of years to the estimated pith location from point #1 (**Table 2**).

**Table 2:** Estimating age of original pith by extrapolation from growth rate between different points on the circumference of Zippori slice (points numbered and dated as in **Figure 2**).

Radius	Likely age range		Distance	ΔYears	Growth rate	Distance of	Estimated
			between	(according	(cm/year)	point #1	year of pith
			points (cm)	to ^14^C)		from pith (cm)	formation
	**Point #1**	**Point #3**					
A	1936–1952	2002–2004	15	50–68	0.2–0.3	11	1900 ± 14
B	1879–1916	2003–2005	13	87–126	0.1	3.5	1868 ± 23
C	1881–1915	2002–2004	14	87–123	0.1–0.2	5	1860 ± 23
	**Point #1**	**Point #2**					
A	1936–1952	1962–1976	9	10–40	0.2–0.9	11	1913 ± 26
B	1879–1916	1954–1955	7	38–76	0.1–0.2	3.5	1869 ± 28
	**Point #2**	**Point #3**					
A	1962–1976	2002–2004	6	26–42	0.1–0.2	11	1881 ± 22
B	1954–1955	2003–2005	6	48–51	0.1	3.5	1868 ± 19

*The likely age ranges for each point along the radii are taken from **Table 1**. The distance in centimeters between all possible combinations of points along a radius (1 to 3, 1 to 2 and 2 to 3) is given, followed by the age difference between both points, according to radiocarbon. The next column describes the growth rate ranges which were obtained as a result of dividing the distance measured between points, by either the maximum or the minimum of the radiocarbon measured time range for the ±1σ calibrated range of each point, resulting in two values for the growth rate, given here as a range for the minimum and maximum growth rate. Given the distance of point #1 to the presumed pith, the number of “missing” years to the pith may be calculated according the growth rate, and an estimate for the time range of pith formation may be obtained. It should be noted that the error range for the estimation of time range of pith formation which is due to uncertainty in pith location estimation is negligible in comparison with the uncertainty due to radiocarbon calibration, and is therefore not taken into consideration here*.

### Growth Rate Calculations

The juvenile phase in olive trees grown from seeds is around 15 years (Santos-Antunes et al., 2005; Vossen, 2007; Fernandez-Ocana et al., 2010). However, olive trees are rarely grown from seeds and are usually vegetatively propagated, shortening the juvenile period to a few years (Vossen, 2007). The difference in growth rate of the juvenile wood was not taken into consideration, and growth rates were considered to be linear. As juvenile wood would be expected to have a larger growth rate, the linearization with non-juvenile wood would cause the pith to be estimated to be slightly older. As we are attempting to determine a *terminus post quem* for the age of the pith, we permit this linearization while noting that the true date for the pith may be later.

Radii A, B, and C seem to have separate piths, indicating the growth of multiple shoots, perhaps meeting at a lower point in the trunk.

#### Radius A

As point A1 was radiocarbon dated to 1936–1952 CE, and point A3 was radiocarbon dated to 2002–2004 CE, the growth rate was calculated for both the shortest time range between the points (2002-1952 = 50) and the longest time range (2004-1936 = 68), and dividing the distance between the two points (15 cm) by each result. The uncertainty in the pith location was found to be negligible compared with the variation due to radiocarbon calibration, and is therefore not presented in calculations. The obtained values for growth rate of radius A were 0.3 and 0.2 cm/year, for each of the time ranges. For calculating pith age, the distance from point A1 to the estimated location of the original pith of radius A (11 cm) was divided by each of the growth rates, resulting in two estimations for the “missing” number of years between point A1 and the original pith. These figures were then subtracted from the date range of point A1, resulting in the estimated year of pith formation at 1900 CE ± 14.

**FIGURE 3:**
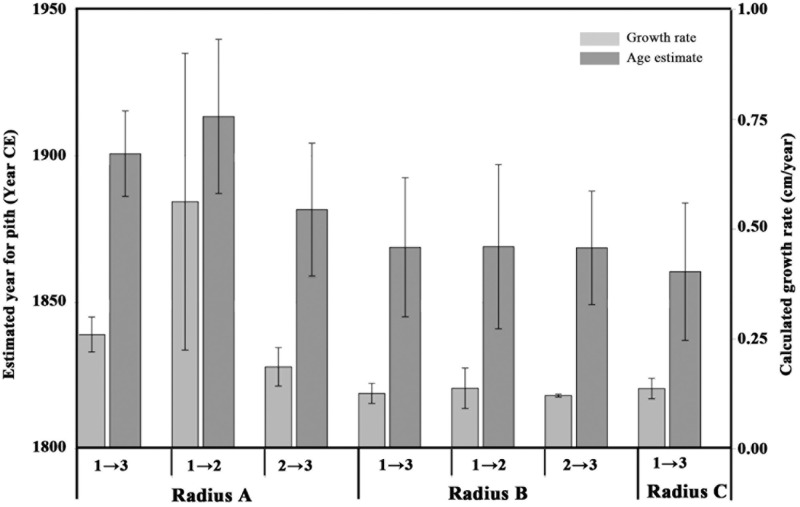
Estimated pith age according to different points and different radii. Light gray bars indicate growth rate (cm per year, axis to the right) calculated between different points on various radii, numbered as in **Figure 2**: point 1 refers to the sampling point closest to the pith; point 3 refers to the sampling point closest to the bark; point 2 is the middle sampling point. Dark gray bars represent estimated ranges for the pith age (years, axis to the left), calculated according to the corresponding growth rate values. The error bars on the light gray bars (growth rate) represent the range between the minimum and maximum growth rate as explained in **Table 2**. The error bars on the dark gray bars (age estimate) represent the range between the minimum and maximum pith age based on the growth rate ranges.

In a similar manner, the growth rate based on the distance between point A1 and A2 (9 cm) and their corresponding radiocarbon dates (1936–1952 CE and 1962–1976 CE, respectively) was estimated to be 0.2–0.9 cm/year. Using this growth rate range, the year of pith formation was calculated to be 1913 CE ± 26. Finally, based on points A2 and A3, the estimated year of pith formation was 1881 CE ± 22. Thus, the pith age ranges obtained by using different combinations of dating points along the radius were comparable (**Figure 3**), although the error range spans nearly 50 years. Points A2 and A3 are both post “bomb peak” (Supplementary Table S1) and therefore the growth rate and the resulting pith date range have a much smaller error range.

#### Radius B

For radius B, growth rates were calculated as for radius A, based on the different combinations of dates and distances between three points (B1, B2, B3, see **Figure 2**). The resulting estimations for year of pith formation were: 1868 CE ± 23 (calculation based on the distance and dating difference between point B1 and B3); 1869 CE ± 28 (point B1 to B2); 1868 CE ± 19 (point B2 to B3) (**Table 2**). Thus the resulting date range for pith formation from radius B are all very similar. However, as was the case for radius A, there is an error range spanning nearly 50 years (**Figure 3**) due to the nature of the radiocarbon calibration curve, as all three points are before the “bomb peak” (Supplementary Table S1).

#### Radius C

As is evident from the cross-section (**Figure 2**), the ring growth along radius C is non-linear. Therefore, we have chosen only the inner and outer points (C1 and C3, respectively) as an exercise in calculating the growth rate between two points while disregarding the pattern observed in cross-section, simulating sampling a live tree where the cross-section cannot be observed. Based on the distance between C1 and C3, and the difference in radiocarbon dates between them, the pith year of formation was calculated as 1860 CE ± 23.

**FIGURE 4:**
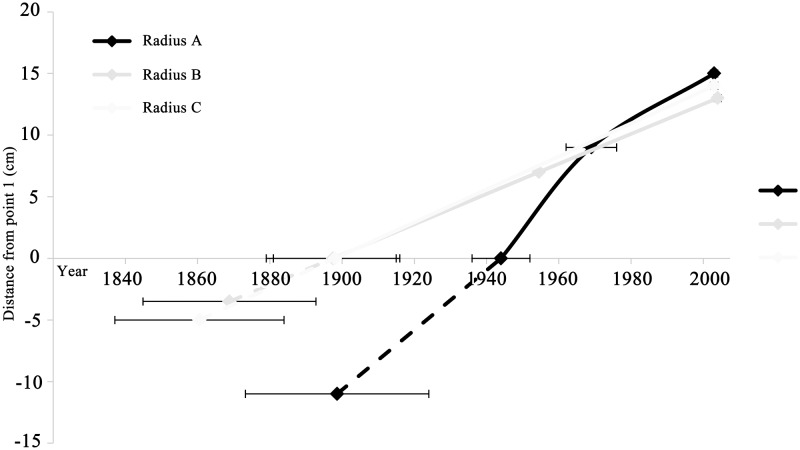
Comparison of growth rates between different radii, represented by the slope of each radius. Although radius C is comprised of only the two extreme points (point 1 and 3), it is shown in order to illustrate the similarity of its slope to radii A and B. Point 1 in all radii is the intercept with the *x* axis. The dashed lines represent the estimated distance from the edge of the remaining wood within the rotted center to the estimated pith location, and the corresponding averaged pith formation year. Point 3 in all radii is the chronologically latest point, while point 2 is the intermediate in radii A and B.

Growth rates were compared between wood closer to the pith (point 1 and 2) and the points in which no juvenile wood is expected (point 2 and 3), for each of the radii A and B (**Figure 4**). While radius B and C have similar growth rates at around 0.13 cm/year, the growth rate of radius A is double that at 0.26 cm/year. Observing the growth rates of radius A reveals a twofold difference: the older wood from point 1 to point 2, perhaps including juvenile wood was calculated to be 0.41 cm/year, while the growth rate of the younger wood, from point 2 to point 3 was about half, at 0.19 cm/year.

## Discussion

Olive trees do not form clear circular annual rings, and the most central wood is frequently rotted. Thus the direct dating of the tree based on ring counting in cores or radiocarbon dating of the pith cannot be carried out. Estimating the age of living olive trees which are thought to be ancient is carried out using indirect methods, such as extrapolating the age based on growth rate estimations. We have found that relying on increase in girth would not be a reliable method for age estimation, as shoots may add to girth disproportionally. Indeed, new trunks may develop around the base of the original tree (Lavee, 1996) and as they grow in close proximity, physical pressure will eventually be exerted between them. This pressure can cause bark breakage, exposing live parenchyma cells, which can create “tissue bridges” eventually forming a continuous cambium ring (Schweingruber, 2007), as is seen in the sub-section in **Figure 2**.

We have shown here that extrapolating the age of an olive tree based on growth rate calculated between points in cross-section may give a rough estimate of the age of the pith. However, as viewing the entire cross-section might not be desirable when a live tree is in question, the distance measured between two selected points may not reflect a true vector of growth, perhaps crossing a number of branches. In addition, growth rates are variable, reduced at the meeting points between independent scaffolds (Lavee, 1996).

The meaning of the calculated result must be considered as well. In the case of a live tree, one pith is usually hypothesized to exist. In olive there are commonly a few, as was the case in the cross-section analyzed here. Assuming the existence of only one central pith could lead to an over-estimation of the age of the tree. In the cross-section analyzed in here, it appears there are three piths, of three different sections of the tree. Thus, the results obtained are of three separate ages, implying one should not combine the three dates when using Bayesian models (like R_Combine in Oxcal), as they do not necessarily represent a single event. Moreover, the sub-section which seems structurally nearly independent of the bulk, is indeed of a distinctly different time (**Figure 2** and Supplementary Table S1).

As is evident from the error range of sampling points from before or during the “bomb peak,” the location in time on the radiocarbon calibration curve is an essential factor in the precision of assessing growth rate. For estimating the age of ancient olive trees, at least one point would be before the “bomb peak,” with an error range that could span 100 years, clearly influencing growth rate calculations. Due to this large range of error due to the radiocarbon calibration, it is difficult to observe whether there could be a difference in the estimated pith age range obtained using different radii or sampling points. Based on the unique view of the entire cross-section in this work enabling an estimation of numbers and patterns of growth ring, it is reasonable to rule out several dates, which otherwise would have been possible based on radiocarbon alone. For example, sample A1 was taken from an area estimated to be ∼77 rings inward from the bark. With a known age of the wood nearest to the bark, and even given a large uncertainty in ring count, it would still be highly unlikely for this sample to be from the 17th century, although this is a probable date range based on radiocarbon. With no knowledge of the internal structure, the probable date for point A1 would have resulted in a deviation of a few centuries from the result we obtained.

Based on our dating results for each point, and considering the probable date ranges, we estimate that the piths are likely dated to 1840–1920 CE. This estimation should be considered as a *terminus ante quem* for the age of the tree, as the main branching out from the trunk occurs during the first years of growth (Ben Sadok et al., 2013).

We have found that the datable wood of the Zippori tree was likely not older than 1879 CE (point B1). This is in agreement with most other existing inner wood within live olive trees which has not been dated to older than 200–300 years, with the exception of unique conditions where trees have been nurtured, such as in Gethsemane (Bernabei, 2015). In addition, radiocarbon dating the cross-section clearly revealed a complex pattern for olive wood growth. As the cross-section is not available for dating live trees, the assumption of linear growth from one central point must be treated with caution.

## Author Contributions

YE carried out the research and experimentation, as part of her Ph.D. LR assisted in research direction and guidance, and carried out the AMS measurements. ZK obtained the sample on which the research was carried out. EB PI of the program and headed the research direction and guidance. All authors read and revised the paper.

## Conflict of Interest Statement

The authors declare that the research was conducted in the absence of any commercial or financial relationships that could be construed as a potential conflict of interest.
