# Supplementary material for: Radiocarbon Dating of an Olive Tree Cross-Section: New Insights on Growth Patterns and Implications for Age Estimation of Olive Trees
Source: Front Plant Sci. 2017 Nov 10;8:1918. doi: 10.3389/fpls.2017.01918 (PMC5686044; doi:10.3389/fpls.2017.01918)
Supplement: Supplementary file 1 [file Table_1.docx]

# Supplementary Material

Table S1. F^14^C of samples from olive branch cross section from Figure 2, and the calibrated dates according to Oxcal (Bronk Ramsey, 2009) and Calibomb (Reimer et al., 2004), using the NHZ2 calibration curve (Hua et al., 2013). All values were corrected according to AMS δ^13^C values which were in the range of botanical C3 material (-20 to -28).

|  | Lab No. | F^14^C | F^14^C +/- 1σ | Oxcal calibration 1σ |
| --- | --- | --- | --- | --- |
| A1 | RT-7373 | 0.97862 | 0.0028 | 1668 (12.7%) 1682  1736 (19.3%) 1759  1760 (19.0%) 1781 1797 (6.1%) 1805  1936 (9.9%) 1947  1951 (1.2%) 1952 |
| A2 | RT-7335 | 1.38099 | 0.0068 | 1962 (11.3%)  1974 (24.9%) 1975  1975 (32.0%) 1976 |
| A3 | RT-7334 | 1.07782 | 0.0029 | 2002 (68.2%) 2004 |
|  |  |  |  |  |
| B1 | RT-7371 | 0.99004 | 0.0031 | 1698 (19.8%) 1722  1816 (13.9%) 1834  1879 (34.3%) 1916  1954 ( 0.2%) 1955 |
| B2 | RT-7370 | 0.99421 | 0.0041 | 1699 (2.6%) 1703  1706 (12.1%) 1720  1818 (11.4%) 1833  1880 (41.3%) 1915  1954 ( 0.7%) 1955 |
| B3 |  | 1.07259 | 0.0029 | 1957 ( 2.6%)  2003 (5.6%)  2003 (59.9%) 2005 |
|  | RT-7331 |  |  |  |
| C1 | RT-7376 | 0.99269 | 0.0032 | 1700 (0.7%) 1701  1707 (12.0%) 1719  1819 (2.5%) 1823  1825 (6.6%) 1833 1881 (45.7%) 1915  1954 ( 0.7%) 1955 |
| C2 | RT-8579 | 0.99506 | 0.0026 | 1892 (64.7%) 1908  1954 (3.5%) 1955 |
| C3 | RT-8577 | 1.07702 | 0.0028 | 2002 (65.2%) 2004  2004 ( 3.0%) 2004 |
| 1 | RT-8597 | 0.99397 | 0.0026 | 1711 (6.0%) 1717  1828 (2.2%) 1831  1891 (58.2%) 1909  1954 ( 1.8%) 1955 |
| 2 | RT-8596 | 1.01226 | 0.0026 | 1955 (68.2%) 1956 |
